# Direct Imaging of Surface Melting on a Single Sn Nanoparticle

**DOI:** 10.1021/acs.nanolett.3c00943

**Published:** 2023-07-07

**Authors:** Aleksandr Kryshtal, Sergiy Bogatyrenko, Olha Khshanovska

**Affiliations:** † AGH University of Science and Technology, Al. A. Mickiewicza 30, Kraków PL-30 059, Poland; ‡ V.N. Karazin Kharkiv National University, 4 Svobody Square, Kharkiv 61022, Ukraine

**Keywords:** nanoparticles, surface melting, quasi-liquid, Sn, in situ transmission electron microscopy, electron energy loss spectroscopy

## Abstract

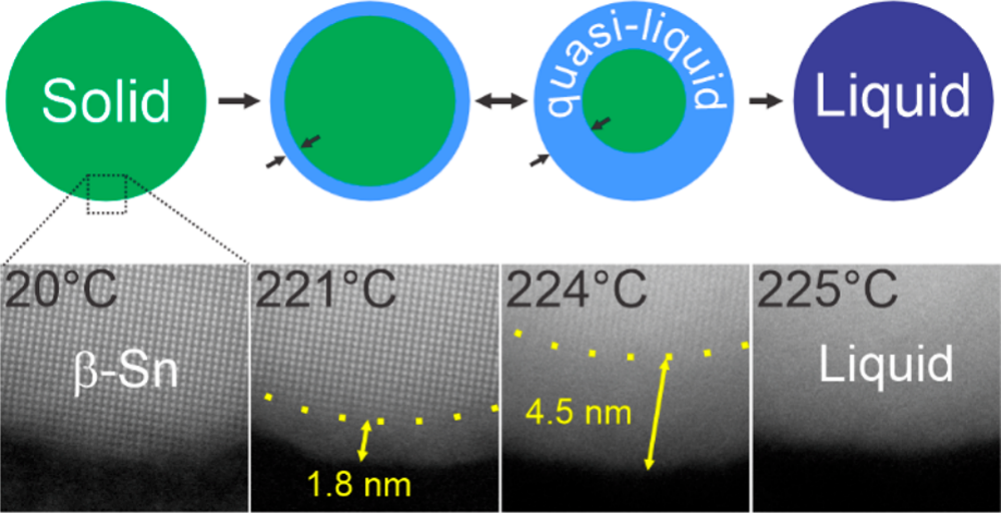

Despite previous studies, understanding the fundamental mechanism of melting metal nanoparticles remains one of the major scientific challenges of nanoscience. Herein, the kinetics of melting of a single Sn nanoparticle was investigated using *in situ* transmission electron microscopy heating techniques with a temperature step of up to 0.5 °C. We revealed the surface premelting effect and assessed the density of the surface overlayer on a tin particle of 47 nm size using a synergetic combination of high-resolution scanning transmission electron microscopy imaging and low electron energy loss spectral imaging. Few-monolayer-thick disordered phase nucleated at the surface of the Sn particle at a temperature ∼25 °C below the melting point and grew (up to a thickness of ∼4.5 nm) into the solid core with increasing temperature until the whole particle became liquid. We revealed that the disordered overlayer was not liquid but quasi-liquid with a density intermediate between that of solid and liquid Sn.

Nanoparticles possess extraordinary physical and chemical properties due to their high surface-to-volume ratio. These properties enabled the wide application of nanoparticles in catalysis, plasmonics, energy generation and storage, electronics, biology, and medicine.^1^ Size depression of the melting temperature of metal nanoparticles is probably the most known phenomenon in nanophysics. It has been studied for more than 100 years^2−15^ since the prediction by Pawlow in 1909,^3^ and we refer the reader to excellent reviews^16−18^ for more detailed information on this topic. However, most of the experimental observations were performed on arrays of nanoparticles, while studies of phase transformations in individual nanoparticles are relatively scarce.^12−15^ As a result, understanding the kinetics of phase transformations in individual nanoparticles remains a scientific challenge. So far, there is no answer to the central question: how does a free nanoparticle melt?

There are three main thermodynamic models of the melting process, which were already extensively discussed in the literature.^10,16−18^ The earliest model was proposed by Pawlow in 1909.^3^ It states that the phase transition from solid to liquid occurs at a single temperature, i.e. the solid nanoparticle melts homogeneously. Another model, which was suggested in 1948 by Reiss and Wilson,^19^ is known as the Liquid Shell Nucleation (LSN) Model. It assumes the formation of a thin liquid layer at the surface of a solid nanoparticle at a low temperature. The liquid layer remains unchanged until the particle melts completely at the melting temperature. And the third model suggests that the nanoparticle surface melts initially, and the liquid layer at the surface grows and moves into a solid as the temperature increases. This is known as the Liquid Nucleation and Growth (LNG) model, and it was proposed in 1977 by Couchman and Jesser.^20^ A vibrational-based model of melting was introduced in the year 1910 by Lindemann,^21^ and it explains the melting phenomenon in terms of instability. Thus, it states that the average amplitude of thermal vibrations of atoms increases with an increase in temperature and melting occurs when the amplitude of vibration exceeds a threshold value (∼10%), which is taken as a fraction of the interatomic spacing in crystals.

All models reasonably fit experimental data and predict a linear dependence of the melting temperature on the reciprocal size of nanoparticles. As a result, the particular mechanism of the melting of nanoparticles remains unclear. Hence, our work aims to unravel the mechanism of melting freestanding metallic nanoparticles.

We used Sn nanoparticles (NPs), which were heated inside a transmission electron microscope (TEM) to observe the kinetics of melting and crystallization in real-time. The NPs were formed on a SiNx substrate kept at room temperature via physical vapor deposition in a vacuum of 10^–7^ mbar. High-temperature annealing (700 °C) was applied after the transfer of the specimen to a TEM to bring the shape of NPs to equilibrium and remove an oxide layer. Two highly complementary techniques were employed for tracing the phase state of the nanoparticle in a heating cycle, namely, high-resolution high-angle annular dark-field (HAADF) imaging and electron energy loss (EEL) spectral imaging (SI) in a scanning transmission electron microscope (STEM). The temperature of the specimen was incremented in steps of 0.5 °C in the premelting region, where we focused our studies. The temperature step was limited by the electron beam heating effect.^22^ More experimental details are available in the Supporting Information. Nowadays, atomic-resolution imaging is a routine technique in a probe Cs-corrected STEM, which enables reliable registration of order–disorder transformations in nanoparticles. At the same time, mapping the liquid and solid states in a single nanoparticle using the EEL SI technique is used here for the first time, to the best of our knowledge.

Figure 1a shows a high-resolution HAADF-STEM image of the Sn NP, which was used in the study. It has an almost spherical shape with a diameter of about 47 nm. The crystallographic structure of the NP corresponds to a single-crystalline β-Sn (tetragonal space group I4_1_/amd with a and c lattice parameters of 0.58 and 0.32 nm, respectively) in the [001] orientation, as follows from the Fourier transformation shown in Figure 1b. A regular, decaying intensity variation toward the surface is seen in Figures 1c and d, revealing an oxide-free surface of the Sn NP. The contact angle of the NP with the SiNx substrate was assessed to 116° (see details in the Supporting Information), which is a typical value for the receding angle of metal nanoparticles on inert substrates.^23^ Inert and nonwetting substrates have a negligible influence on the melting temperature of nanoparticles;^24^ therefore, a Sn nanoparticle on SiNx could be considered as a nearly free one.

**Figure 1:**
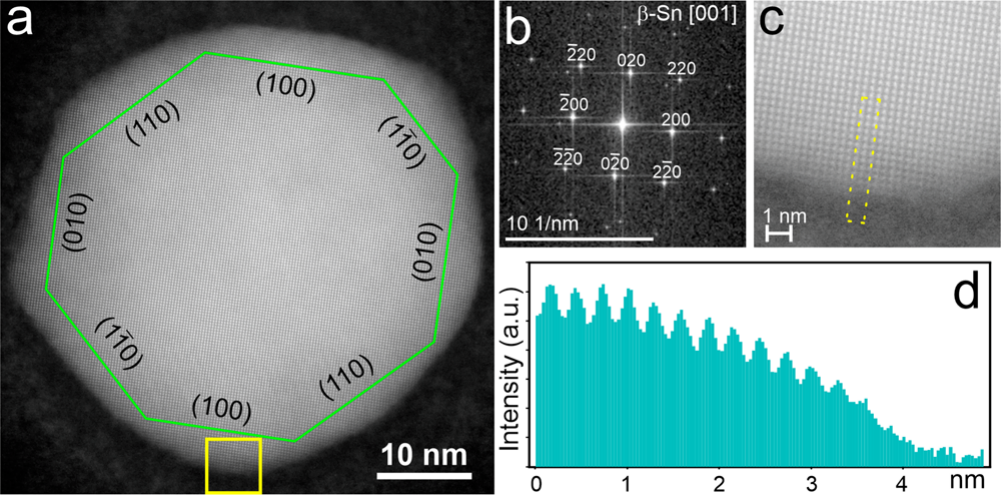
(a) High-resolution HAADF STEM image of β-Sn NP at room temperature along with (b) its Fourier spectrum. (c) Zoom-in view of the yellow square region in a, and (d) a spatially averaged intensity profile across the selected area in c.

Figure 2 presents selected HAADF STEM images of this NP during heating. The extended set of data is available in the Supporting Information (Figure S2). The Sn NP remained solid up to a temperature of ∼200 °C. At this temperature, melting starts with the formation of a thin disordered layer at the surface. Its thickness was not constant and varied over the surface of the NP. Thus, the largest thickness of ∼0.8 nm was registered in the surface region with the highest curvature (arrow in Figure 2a), while the thickness of the disordered layer in the rest of the surface did not exceed 0.4 nm or 1–2 atomic layers (Figure 2a′). The disordered layer almost exponentially expanded to the core of nanoparticle as the temperature increased from 200 to 224 °C (Figure 2a–d, and Figure S3 in the Supporting Information) and its thickness reached 4.5 nm in the zoomed-in region of the NP (Figure 2d′) just before complete melting. The width of the order–disorder interface, which was assessed from the intensity fluctuations decay in the high-resolution images, was about 3 atomic layers or ∼0.8 nm.

**Figure 2:**
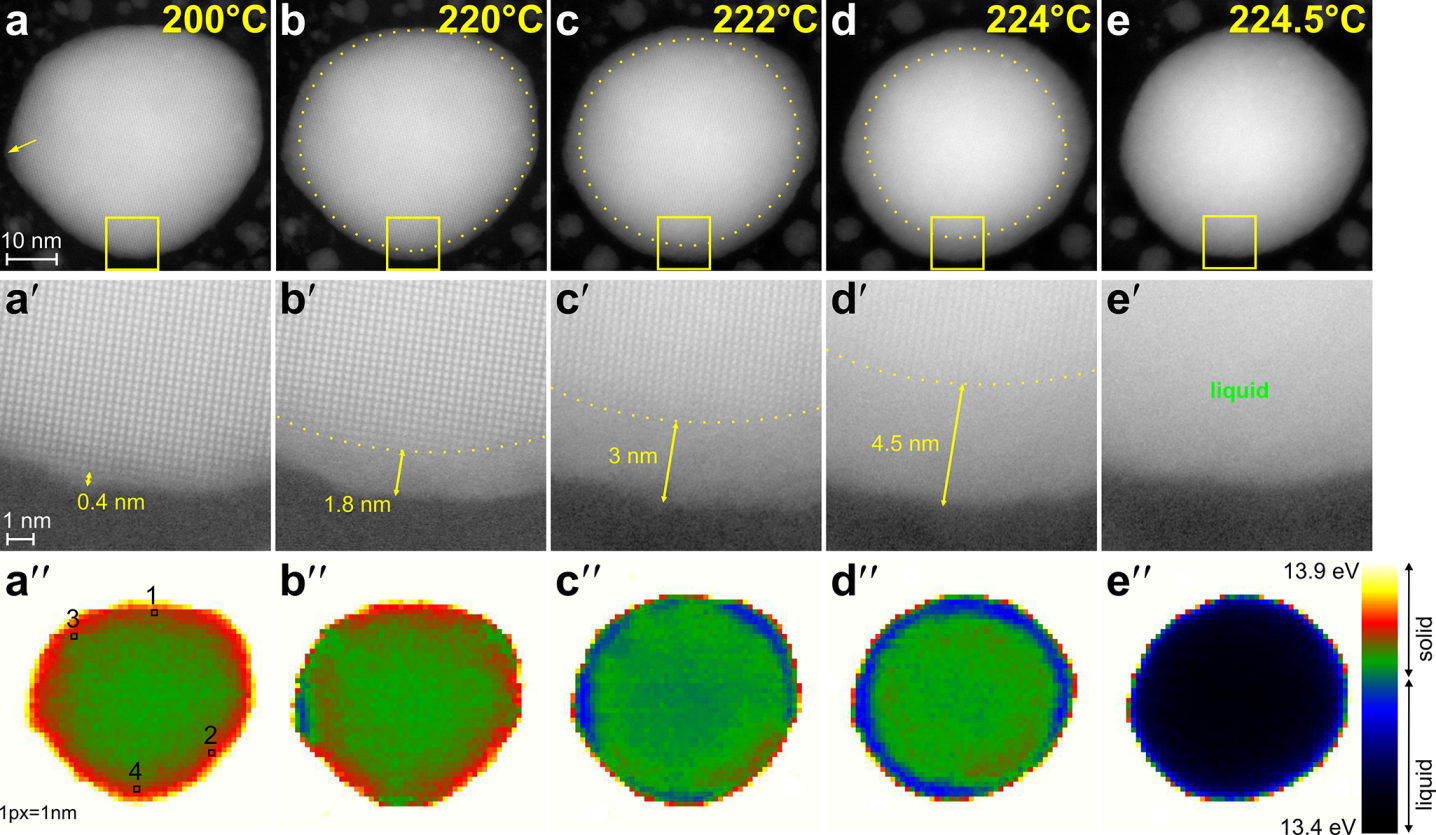
(a–e) HAADF STEM images and (a″–e″) false-color plasmon peak energy maps of the same Sn nanoparticle at different temperatures. The temperature is specified in the top right corner of the images. (a′–e′) Zoom-in images showing the particle surface region marked with a yellow rectangle in images a–e. The order–disorder interface is shown with dotted lines in a–e and a′–e′. The scale bar is the same for all images in the row.

Importantly, the disordered layer at the surface of Sn NP is an equilibrium one, which was proved by two observations. First, its thickness did not change over ca. 15 min of observation. Second, the thickness of the layer decreased with the temperature reduction resulting in epitaxial crystallization of the disordered phase without a noticeable undercooling (see Figure S4c–f in the Supporting Information). In other words, the order–disorder transformation is reversible. This is in line with observations of surface melting of Sn nanoparticles embedded in SiO_2_ matrix.^25^

The complete melting of the Sn nanoparticle occurred at ∼224.5 °C which is revealed by a disappearance of the crystalline lattice in the core of the Sn NP (Figure 2e).

We repeated the study for several Sn nanoparticles, and their melting behavior had the same pattern, which justifies the general nature of the effect (see the Supporting Information for more details). As can be seen, the melting transition of the Sn NPs takes place over a temperature range of about 25°. At the same time, nanocalorimetric measurements^26,27^ showed that the melting of Sn NPs initiates 60–80° below the melting point. The discrepancy is probably due to the integral nature of nanocalorimetric techniques, resulting in data averaged over an array of nanoparticles with a wide size distribution.

It is natural to assume that the disordered layer that was observed at the surface of the Sn nanoparticle is a liquid one. Nevertheless, as will be shown below, the disordered overlayer turned out to be not a liquid but quasi-liquid.

Melting is the first-order phase transformation, which has two distinct features.^28^ First, the latent heat of the transformation is nonzero. Second, an abrupt change in the volume occurs. And namely, the volume (or density) change under melting and crystallization could be registered by the EELS technique in a TEM via a shift of the volume plasmon peak position in the low-loss region of the spectrum.^29,30^ Indeed, the energy of bulk plasmon oscillations is specific for each material and within the Drude model is defined by equation^31^
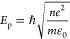
1where *ℏ* is the Planck constant, *n* is the density of valence electrons, *e* and *m* are the electron charge and mass, respectively, and ε_0_ is the vacuum dielectric constant. The density of valence electron is proportional to the density of material ρ
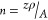
2where *z* is the number of valence electrons per atom, and *A* is the atomic weight.

Figure 3 shows low-loss spectra from the center of solid and liquid Sn NP with a diameter of ∼45 nm (inset in Figure 3b) along with the temperature dependence of the plasmon peak energy *E*_p_ in a heating–cooling cycle. The volume plasmon energy of the Sn NP amounted to ∼13.75 eV at room temperature, which is consistent with reference data of tetragonal Sn (13.7 eV).^31^ A slight linear decrease of the E_p_ occurred as the temperature increased in the range 20–225 °C, which is mainly due to the volumetric thermal expansion of solid Sn. The calculated slope was −0.3 meV/°C. An abrupt change in the free electron density occurred at melting at a temperature of *T*_m_ ≈ 225 °C (Figure 3). The value of volume change of the Sn NP on melting amounted to −3.2%, which is slightly higher than the reference one of Sn (−2.3%).^32^ The crystallization of liquid Sn NP occurred with a large overcooling at a temperature *T*_g_ ∼ 100 °C and resulted in a retrieval of the *E*_p_ value to that of solid Sn. The relative value of overcooling upon crystallization  amounted to 0.25, which is an expected one for metallic NPs having a contact angle of 116° with amorphous substrates.^33^ It is worth noting that the melting-crystallization temperature hysteresis was fully reproducible on the same Sn NP in several thermal cycles, revealing the consistency of the data acquired.

**Figure 3:**
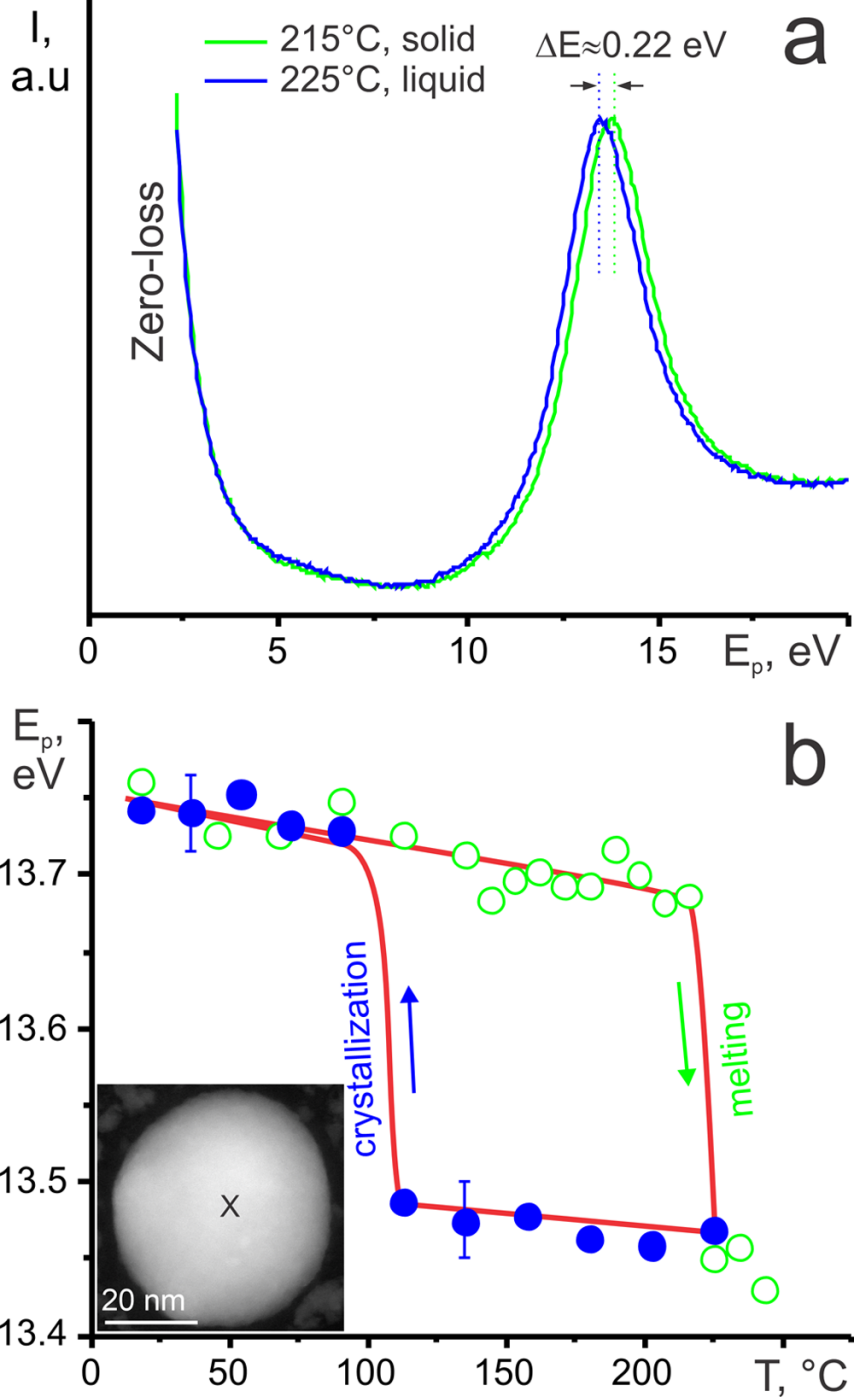
(a) Low-loss EELS spectrum of solid and liquid Sn nanoparticles and (b) volume plasmon peak energy *E*_p_ of Sn nanoparticle versus temperature. Open circles correspond to heating and close circles to the cooling cycle. Inset shows a HAADF STEM image of liquid Sn nanoparticle under study with a cross marking the region of data acquisition. A solid line is included to emphasize the melting-crystallization hysteresis.

A relatively large difference in *E*_p_ between solid and liquid states enabled us to map these states in individual Sn nanoparticles during heating using the EEL spectral imaging (SI) technique. Figure 4a shows the plasmon peak energy map of the Sn NP under study at room temperature. The energy values were coded by a false color scale to reveal minor variations. Plasmon peak energy profile across the radius of the NP shows that its value was almost constant over the core of the NP (Figure 4b) and amounted to ∼13.8 eV. At the same time, plasmon peak energy gradually increased to ∼14 eV when approaching the surface of the NP, which is likely due to size and quantum confinement effects.^34^

**Figure 4:**
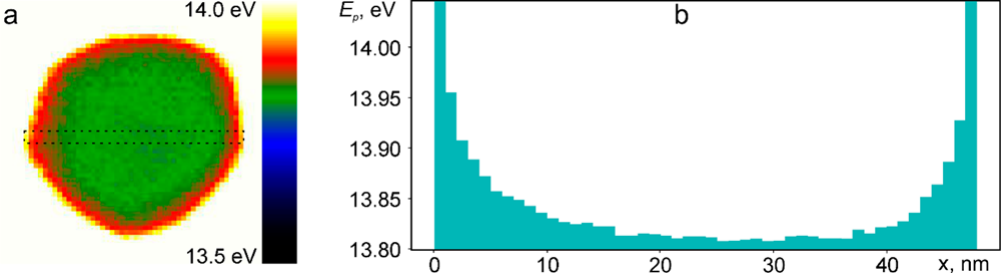
(a) Plasmon peak energy map of Sn nanoparticle and (b) its profile across the selected region.

Figure 2a′′–e′′ shows the plasmon peak energy map of Sn NP under study in the temperature range 200–225 °C. The solid is coded with green and red color (13.68–13.9 eV), while the liquid/disordered state is coded with black and blue color shades (13.4–13.68 eV). Such separation on ordered and disordered states with a boundary at 13.68 eV followed from the data of Figures 3b and 4b and is somewhat artificial. It is aimed to distinguish phase transformations and edge effects in spectral images. It can be seen from Figure 2a′′ that the nanoparticle was fully solid at 200 °C, i.e., the plasmon peak energy did not fall below a threshold value of 13.68 eV in any part of the Sn NP. The first liquid was registered at the surface of the NP at 220 °C (Figure 2b”). The fraction of the surface with a liquid layer grew as the temperature increased to 222 °C, and at a temperature of 224 °C, almost the entire nanoparticle was surrounded by a liquid layer. Finally, the core of Sn NP melted at a temperature of 225 °C, which is evident from Figure 2e′′.

Hence, our observations revealed that the surface of Sn NP melts initially, and the equilibrium disordered/liquid layer grows and spreads into the solid with an increase in temperature. As a result, the liquid nucleation and growth mechanism of Couchman and Jesser^20^ seems the most relevant thermodynamic approach for nanoparticle melting.

It is noteworthy that both EELS SI and HAADF-STEM studies revealed that the nucleation of the disordered layer was not uniform over the surface of the NP. We are confident that the local curvature and crystallography of the surface are the main factors influencing the nucleation of the liquid phase. Thus, the least close-packed crystallographic surfaces with the highest surface energy melt first, while those with the lowest surface energy did not premelt or exhibit a late premelting effect.^35^ Unfortunately, we were not able to reliably identify preferable Sn facets in this study, and a reconstruction of the NP shape in 3D is required for a further insight.

Apart from spectral images, EELS data deliver quantitative information on the free electron density across the order–disorder interface of a partly molten Sn nanoparticle, although the accuracy of such data is moderate. It was methodologically convenient to trace the temperature dependence of the *E*_*p*_ in selected points of the nanoparticle rather than study the peak energy profile across the order–disorder interface at a particular temperature. Figure 5 shows the relative density change  of Sn, which was calculated from *E*_p_ data, in edge points #1–4 and the center of the Sn nanoparticle (Figure 2a”) in the vicinity of the melting temperature. Points #1, 3, and 4 represent the Sn surface exhibiting the premelting effect. Point #2 is the part of the Sn surface, which did not premelt according to EELS data (Figure 2d′′) but showed a thin disordered layer in high-resolution images at a temperature of 224 °C (Figure 2c, d).

**Figure 5:**
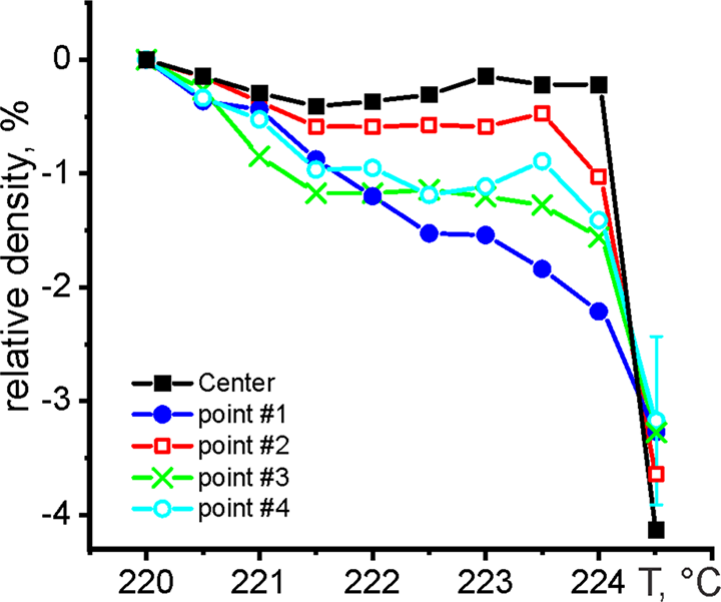
Relative density change at points #1–#4 (Figure 2a′′) and at the center of Sn NP versus temperature. The error bars are the same for all data points.

The pattern of the temperature change of density for point 2 was almost similar to that observed in the center of Sn NP (Figure 5). Thus, it slightly decreased as the temperature increased followed by a sudden fall at the melting point of Sn NP of 224.5 °C. However, a 1% drop in the density was registered just before melting at a temperature of 224 °C. This change in the density was sufficient to form a disordered structure, which was observed in the high-resolution image of Figure 2d.

At the same time, the pattern of the change in density for points 1, 3, and 4 differs substantially. Most importantly, it revealed a quasi-liquid state of the disordered layer instead of a liquid one, as supposed in thermodynamic models of surface melting.

Thus, the temperature dependence of the density change in points #3 and 4 showed a distinct plateau at −1% followed by a gradual decrease to a value characteristic of the liquid phase. Hence, the density of the disordered phase was almost constant over the 221.5–223.5 °C temperature range, and its value was ∼1% lower than that of solid. This change is too small to treat the disordered layer at the surface of the Sn NP as a liquid phase. The density in point #1 showed almost a monotonic decrease in the temperature range of 220–224 °C. We suppose that several facets contribute to a signal at this point, resulting in its averaging. Nevertheless, the density in the disordered layer at the surface of a partly molten Sn nanoparticle reached that of the liquid phase only at the melting point of Sn NP.

The long-time stability of the disordered overlayer and the revisability of order↔disorder transformation convinced us that the disordered layer is not an amorphous phase. Indeed, amorphous is a metastable phase, and its transition to an equilibrium state (liquid or crystal) requires overcoming an energy barrier. It is very unlikely that a decrease in the temperature of ∼1° ensures sufficient energy gain for that. Even assuming that this is the case, the entire amorphous overlayer must crystallize since the latent heat released at the crystallization front will make the process self-sustaining at this temperature. This was not observed experimentally, and the disordered overlayer remained stable. Therefore, we called the disordered layer at the surface of Sn nanoparticles a quasi-liquid, which is an intermediate state between a solid and an ordinary bulk liquid. It is worth noting that the viscosity of the liquid layer in partly molten nanoparticles intermediate between typical values for liquid metals and glasses,^25^ which is in line with our observations. The formation of a quasi-liquid layer that expanded to the core with increasing temperature was reported in molecular dynamics simulation of the surface melting of Ag nanoparticles.^36^ Frozen water has a quasi-liquid layer at its surface that exists even well below the bulk melting temperature.^37^ Nevertheless, the existence and properties of a quasi-liquid on free crystal surfaces are poorly understood and lack a general theory.^37,38^

Lindemann’s vibrational instability approach seems the most appropriate model among semiempirical approaches for describing the quasi-liquid layer. Indeed, the mean square displacement of Sn atoms depends on the temperature and distance from the surface, and when the average amplitude of vibration reaches a threshold value, the disordered layer is observed. Even though the amplitude of thermal vibrations increases with increasing temperature, the solid core “holds” the atoms of the disordered shell, preventing the formation of a “true” liquid. At the same time, the presence of a plateau for graphs of points 2–4 in Figure 5 indicates that the properties of the quasi-liquid layer, in the first approximation, remain uniform over the layer. Therefore, the quasi-liquid layer could be treated as a separate phase, and the order–disorder transformation in the surface layer is a discontinuous one; i.e., it can be considered a first-order transformation. Hence, the thermodynamic model of surface melting remains valid for the disordered layer, as well. Surface melting is possible when the sum of the free energies of the solid core and its liquid layer is lower than the free energy of the free solid surface:^25^
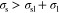
3where *σ*_s_ and *σ*_l_ are the surface energy of solid and liquid phases, correspondingly, and *σ*_s1_ is the solid–liquid interphase energy. The surface energy of the disordered and liquid phases should be comparable and lower than the solid one due to the lack of the long-range order of crystalline solids. Hence, condition (3) is fulfilled for the disordered phase at the surface of a nanoparticle.

Our results thus provide novel insight into the basic properties of nanoparticles in the vicinity of the melting point. Beyond their fundamental importance for materials science and chemistry, they are crucial for catalysis, sensors, electronics, and environmental and biomedical fields, where nanoparticles are widely used and their stability and performance under various conditions are key parameters.

In summary, we revealed the surface premelting effect on a single Sn nanoparticle using advanced TEM techniques. The effect became significant only in the temperature range ∼3° below the melting point, and the thickness of the disordered overlayer reached ∼0.1 of the nanoparticle diameter just before complete melting. At the same time, we found that the disordered overlayer was not an ordinary liquid but a quasi-liquid. Its density is intermediate between solid and liquid Sn. The nucleation and growth of the quasi-liquid layer as well as its density were not uniform over the surface of the NP, which is likely due to a surface anisotropy of Sn nanoparticles. The efficiency of the valence EELS approach for studying phase transformations in single nanoparticles was shown.

## Supporting Information Available

Experimental details, radiation damage in STEM, assessment of the wetting angle of the Sn nanoparticle, extended data set, reproducibility of the effect (PDF)
