# Supplementary material for: Direct Imaging of Surface Melting on a Single Sn Nanoparticle
Source: Nano Lett. 2023 Jul 7;23(14):6354–9. doi: 10.1021/acs.nanolett.3c00943 (PMC10375590; doi:10.1021/acs.nanolett.3c00943)
Supplement: Supplementary file 1 — nl3c00943_si_001.pdf [file nl3c00943_si_001.pdf]

Supporting Information for

Direct imaging of surface melting on a single Sn nanoparticle

Aleksandr Kryshtal[a], Sergiy Bogatyrenko[b], Olha Khshanovska[a]*

[a]AGH University of Science and Technology, Al. A. Mickiewicza 30, PL-30 059 Kraków, Poland

[b]V.N. Karazin Kharkiv National University, 4 Svobody sq., 61022 Kharkiv, Ukraine

* Corresponding author: kryshtal@agh.edu.pl

Contents

Experimental details 1

Radiation damage in STEM 2

Assessment of the wetting angle of the Sn nanoparticle. 3

Extended data set 3

Reproducibility of the effect 5

Literature 7

Experimental details

The nanoparticles were formed by the thermal deposition of Sn island film on the SiN membrane of a MEMS (Microelectromechanical systems) chip (Wildfire$_{HB}$ GT, DENSsolutions). The mass thickness of the film was 10 nm, which was measured by quartz crystal microbalance, and the deposition was performed in a vacuum of $2x10^{-7}$ Torr on the substrate at room temperature.

Sn nanoparticles were annealed at a temperature of 700 °C for about 5 minutes to remove a surface oxide layer, which was formed during a transfer of the sample from the vacuum chamber to a TEM. Liquid Sn has a vapor pressure of ~10^{-8} Torr at 700 °C [1], which is comparable with the column vacuum of a TEM, however, the specimen was retracted from the pole piece of the objective lens for annealing, for safety reasons.

The particle for the study was chosen according to the following criteria. First, the nanoparticle should be free-standing to ensure the conservation of matter during thermal cycles. Second, its size should fit the range of 30-80 nm to ensure high-resolution (HR) imaging and exclude the effect of size. And finally, the orientation of the nanoparticle to a low-index zone axis should be possible within a few degrees of sample tilting.

For the characterization of nanoparticles, we used FEI Titan G2 cubed 60-300 probe Cs-corrected TEM operated at 300 kV, equipped with a GIF Quantum SE System (Model 963, Gatan). The microscope was fitted with a double-tilt MEMS-based heating holder (Wildfire D6 from

DENSsolutions) for *in situ* studies. The temperature readout of the heating holder was calibrated using a melting point of 100 nm-sized Sn nanoparticle of 232 °C.

High-quality HR STEM images were created by averaging 20 frames of the rapid scan image (2048x2048 pixels in size, 200 ns pixel dwell time) by using the commercial software, Velox™ (Thermo Fisher Scientific Inc.) at each temperature. This is favored over a single slow scan image with a long acquisition period because of a better signal-to-noise ratio and lower scan noise. A beam current of 100 pA, convergent semi-angle of 25 mrad, and high-angle annular dark-field (HAADF) detector collection angle of 50–200 mrad were used. Therefore, the total acquisition time for each HAADF STEM image was 25.8 sec, which corresponded to the effective dose of 4×10^6 e^-/nm^2.

The low electron energy loss (EEL) spectral images (SI) 55 x 55 pixels in size were acquired with a pixel size of 1 nm, binning x1, a dwell time of 0.1 s, and 16 x 16 subpixel scanning. In the EEL SI technique, the focused electron beam scans over a selected region of the sample, and the low-loss EEL spectrum is acquired at each raster point, resulting in three-dimensional data, two spatial and one spectral dimension. Spatial drift correction was applied every 5 lines of the scan. Total SI acquisition time was 13 min 44 sec, resulting in an electron dose of 5×10^7 e^-/nm^2 per map. A collection semi-angle of 19 mrad was used. The energy spread of the electrons was approximately 1.1 eV, as measured from the full width at half-maximum (FWHM) of the zero-loss peak.

In each point of the 3D spectra data set, the EEL spectrum was aligned at the maximum zero-loss peak intensity with subpixel accuracy using HyperSpy routine. Then, the energy of the Sn volume plasmon peak was fitted with the Lorentzian function and measured using a Phyton code for HyperSpy library [2] for unbiased data. The energy window 11-17 eV was used for analysis. The plasmon peak energy of the SiNx substrate is ~23 eV, which is far away from the peak of Sn (13.75 eV). Therefore, the plasmon peak of the substrate contributed only to the background of the Sn plasmon peak, which was taken into account in the fitting procedure. Finally, a map of the plasmon peak energy for Sn NP was created for each temperature studied.

The accuracy of the measurement was limited by the dispersion of the spectrometer and was assessed to be ±0.05 eV. At the same time, the precision, which is the standard deviation in a set of repeated measurements, did not exceed ±0.01 eV for data of Fig. 2. The precision was assessed assuming the plasmon peak energy is constant in the core of the nanoparticle (Fig. 4b).

Radiation damage in STEM

The electron radiation did not fundamentally influence the melting of Sn nanoparticles under illumination parameters used in the study. Thus, the displacement threshold of crystalline Sn for electron irradiation is much higher than the primary beam energy of 300 keV [3]. The sputtering rate of Sn that was calculated according to Egerton [4] for most demanding EELS experiments was ~2 monolayers/s, which is acceptable for a pixel dwell time of 0.1 sec. The local temperature increment induced by the electron beam in STEM mode did not exceed 0.5 °C for NPs on SiNx substrate [5].

Assessment of the wetting angle of the Sn nanoparticle.

One of the parameters for characterizing the nanoparticle-substrate system is the contact angle θ. Herein, the contact angle was assessed from a plain-view HAADF STEM image of a liquid Sn nanoparticle (Fig. S1b) using the photometric method [6].

In HAADF STEM imaging the intensity of scattering electrons I is proportional to the sample thickness h and the atomic number of the element Z

$$I \sim h \cdot Z^{\alpha}$$

where α - is a coefficient between 1.6 and 2 [7].

The thickness of the Sn nanoparticle has two distinct regions along the diameter (Fig. S1a). Thus, the thickness in region I varies significantly, while the thickness in region II changes in a narrow range only. Therefore, the slope of the first derivative $\frac{dI}{dx}$ has an inflection at the triple point, which allows one to determine its position. Figure S1c shows the intensity profile across the diameter of the Sn nanoparticle and its derivative $\frac{dI}{dx}$. The position of inflection points is marked with dotted red lines. Therefore, the diameter of the contact area d was determined from the graph (Fig. S1c), while the radius of the nanoparticle R was measured directly from the image (Fig. S1b).

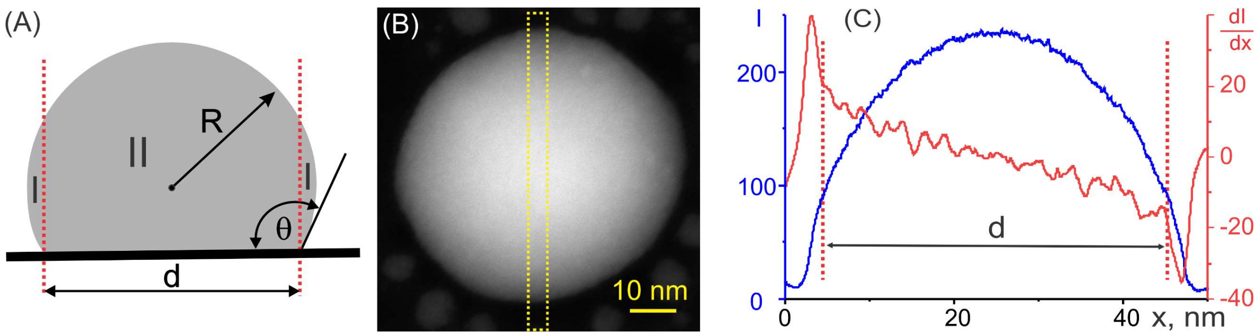

Figure S1. *(A) A Schematic view of nanoparticle cross-section on a solid substrate, (B) HAADF STEM image of liquid Sn nanoparticle. (C) The spatially averaged intensity profile (blue) across the region, marked by a yellow rectangular in (B), and its derivative (red).*

Assuming the spherical shape of the nanoparticle, the contact angle θ was calculated by using the following equation:

$$\theta = 180° - \arcsin\frac{d}{2R}, \qquad \theta > 90°$$

We performed the measurements of d in Fig. S1b along the x and y axis and at an angle of 45 degrees. The assessed value of the contact angle for the nanoparticle varied in the range of 110 – 120°, with a mean value of 116°.

Extended data set

Figure S2 presents extended data set for the Sn nanoparticles, shown in Figure 2 of the main text. Results of the measurements of the thickness of the disordered layer in the bottom part of the Sn NP according to HAADF STEM images of Figure S2 are shown in Figure S3.

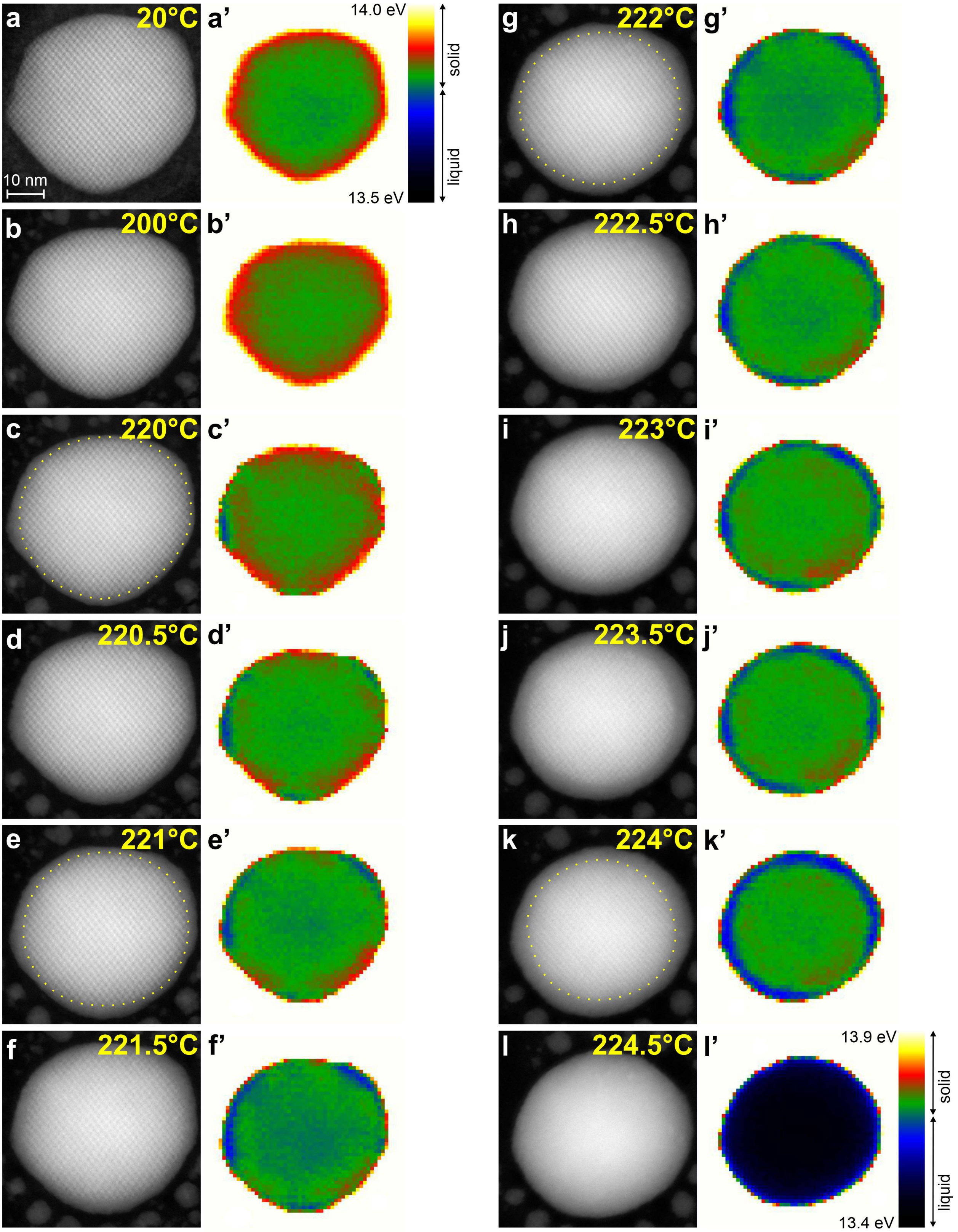

Figure S2. *Extended set of HAADF STEM images (a-l) and false-color plasmon peak energy maps (a'-l') of the very same Sn nanoparticle at different temperatures. The temperature is specified in the top-right corner of the images. The order-disorder interface is shown with dot lines. The scale bar is the same for all HAADF STEM images.*

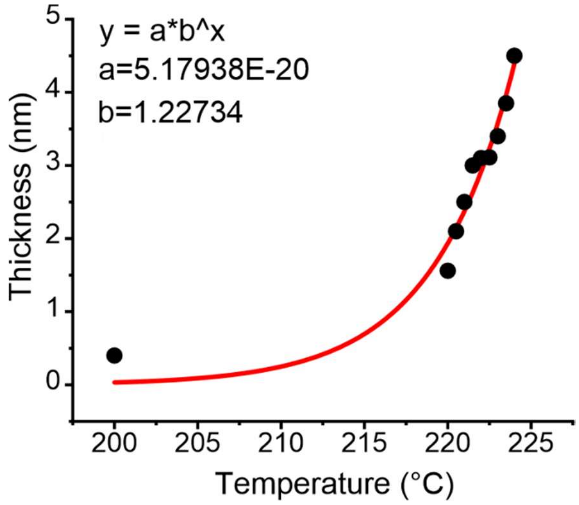

Figure S3. *The thickness of the disordered (quasi-liquid) layer during heating of 47 nm Sn nanoparticle. A red line is the exponential data fit.*

Reproducibility of the effect

Figures S4 and S5 present data acquired for another Sn nanoparticle in consequent heating-cooling cycles. In the first cycle, high-quality HAADF STEM images were acquired, while in the second one, we focused on EELS studies.

The first thermal cycle was divided into 4 parts. Firstly, the Sn nanoparticle was gradually heated to 223°C (Fig.S4 a-d). The temperature was then decreased to 222°C, followed by heating up to a melting point of 226°C. And in the final step, the nanoparticle was slowly cooled down to room temperature.

The disordered layer had a thickness of 0.66 nm at a temperature of 220°C and at 224°C it grew to 1.5 nm (Fig. S4d). The thickness of the disordered layer decreased to 1.1 nm when the temperature decreased to 222°C (Fig. S4e). A subsequent increase in temperature to 225°C led to the regrowth of the disordered layer to 1.5 nm (Fig. S4f). Importantly, the thickness of the disordered layer follows the change in the temperature of the Sn nanoparticle. Please note that the orientation of the layer remained unchanged under order↔disorder transition. The nanoparticle melted completely at 226°C (Fig. S4g). After recrystallization, it was oriented to the nearest zone axis, and its atomic structure is shown in Fig. S4e. It corresponded to β-Sn in the [111] orientation.

Figure S5 shows the plasmon peak energy map of the very same Sn nanoparticle in the second thermal cycle. EELS SI maps (39 x 42 pixels in size) were acquired with a pixel size of 1 nm, binning x5, a dwell time of 0.05 s, and a collection semi-angle of 4.9 mrad. Total SI acquisition time was 1 min 32 sec. Excitation of the TEM monochromator ensured an electron energy spread of 0.45 eV, as measured from FWHM of the zero-loss peak in the EELS spectra.

The liquid layer, which was detected at the surface of the Sn nanoparticle at 224°C (Fig. S5c), gradually expanded to the core of the nanoparticle as the temperature increased till the entire nanoparticle became liquid. Upon cooling, the nanoparticle crystallized far from a zone-axis, in this case; however, the EELS map gave the plasmon peak energies characteristic to β-Sn (Fig. S5f).

Though the general pattern of melting in the second cycle (Fig. S5) was in line with the observations in Fig. S4, the liquid phase nucleated in other parts of the Sn nanoparticle. This is because the liquid nanoparticle crystallizes in a random orientation in every thermal cycle,

resulting in a different crystallography of the surface. This observation supports our statement that crystallography of the surface of Sn nanoparticles controls the surface melting phenomenon.

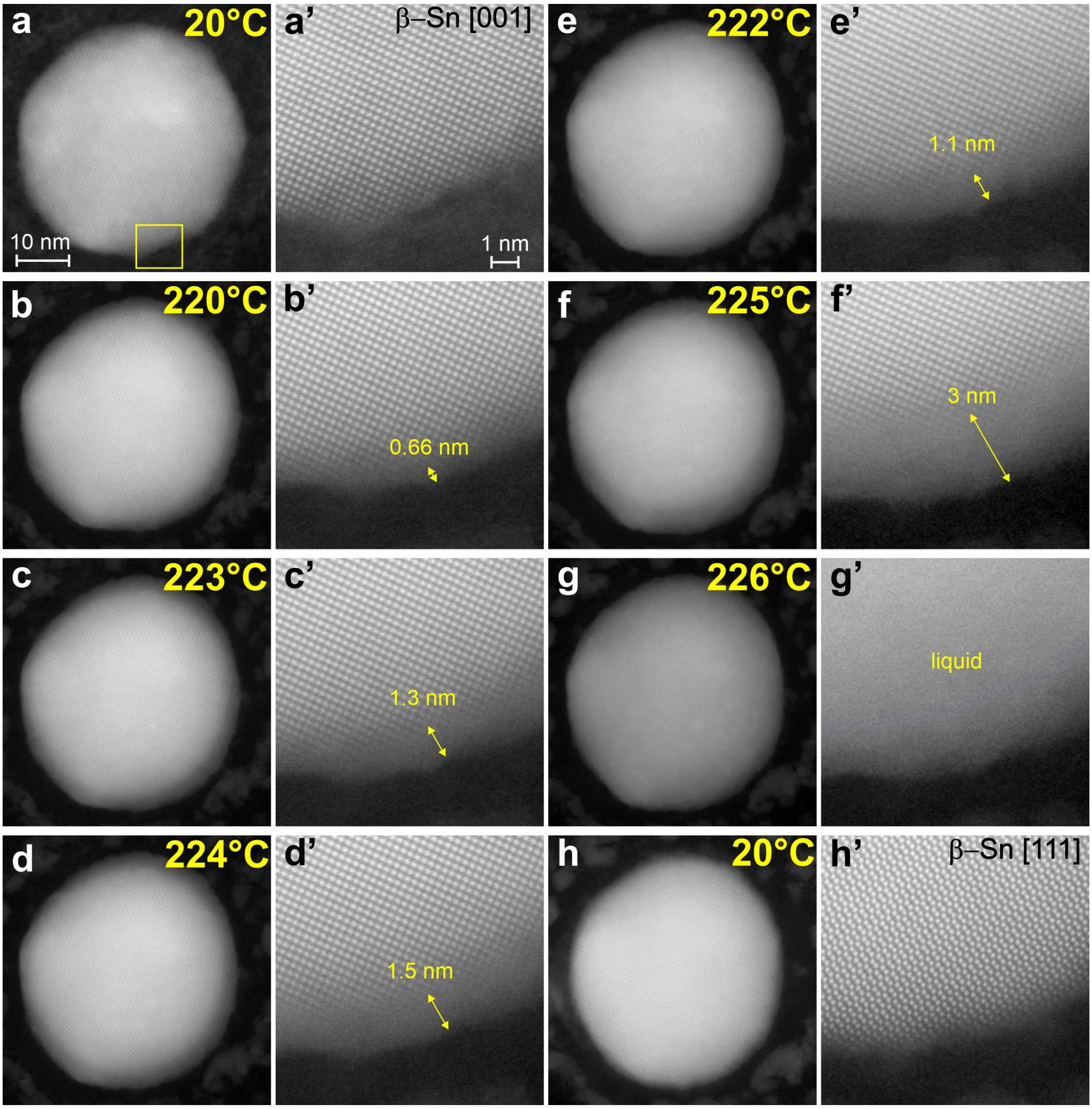

Figure S4. *(a-h) HAADF STEM images of the very same Sn nanoparticle in a heating-cooling cycle. (a'-e') shows zoom-in region marked with yellow rectangular in (a). The temperature is specified in the top-right corner of the images. The scale bar is the same for all images in the column. Please note that the temperature was decreased in (e) followed up by re-heating in (f and g).*

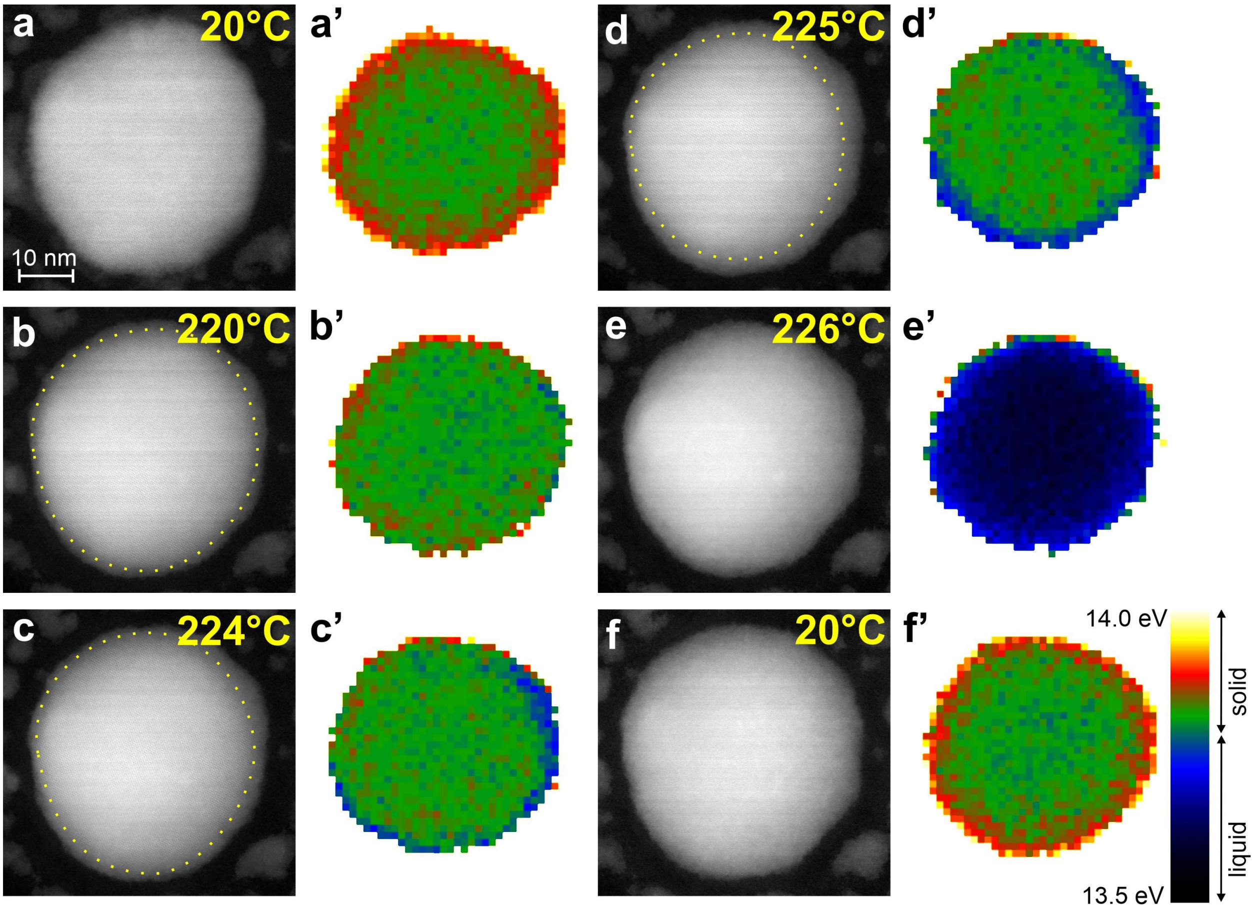

Figure S5. *HAADF STEM images (a-f) and false-color plasmon peak energy maps (a'-f') of the same Sn nanoparticle at different temperatures. The temperature is specified in the top-right corner of the images. The scale bar is the same for all images in the row. The pixel size is 1 nm in EELS maps. The order-disorder interface is shown with dot lines.*

Literature

(1) Alcock, C. B.; Itkin, V. P.; Horrigan, M. K. Vapour Pressure Equations for the Metallic Elements: 298-2500k. *Canadian Metallurgical Quarterly* **1984**, *23* (3). https://doi.org/10.1179/cmq.1984.23.3.309.

(2) Peña, F. de la; Prestat, E.; Fauske, V. T.; Burdet, P.; Lähnemann, J.; Jokubauskas, P.; Furnival, T.; Nord, M.; Ostasevicius, T.; MacArthur, K. E.; Johnstone, D. N.; Sarahan, M.; Taillon, J.; Aarholt, T.; pquinn-dls; Migunov, V.; Eljarrat, A.; Caron, J.; Francis, C.; Nemoto, T.; Poon, T.; Mazzucco, S.; actions-user; Tappy, N.; Cautaerts, N.; Somnath, S.; Slater, T.; Walls, M.; Winkler, F.; Ånes, H. W. Hyperspy/Hyperspy: Release v1.7.3. **2022**. https://doi.org/10.5281/ZENODO.7263263.

(3) Hobbs, L. W. Radiation Effects in Analysis of Inorganic Specimens by TEM. In *Introduction to Analytical Electron Microscopy*; Springer US: Boston, MA, 1979; pp 437–480. https://doi.org/10.1007/978-1-4757-5581-7_17.

(4) Egerton, R. F.; Li, P.; Malac, M. Radiation Damage in the TEM and SEM. *Micron* **2004**, *35* (6), 399–409. https://doi.org/10.1016/j.micron.2004.02.003.

(5) Kryshtal, A.; Mielczarek, M.; Pawlak, J. Effect of Electron Beam Irradiation on the Temperature of Single AuGe Nanoparticles in a TEM. *Ultramicroscopy* **2022**, *233*. https://doi.org/10.1016/j.ultramic.2021.113459.

(6) Gladkikh N.T.; Chizhik S. P.; Larin V. I.; Grigoryeva L. K.; Dukarov S. V. Methods of Determination of Wetting in Highly Dispersed Systems. *Phys. Chem. Mech. Surfaces* **1987**, *4* (11), 3465–3482.

(7) Hartel, P.; Rose, H.; Dinges, C. Conditions and Reasons for Incoherent Imaging in STEM. *Ultramicroscopy* **1996**, *63* (2), 93–114. https://doi.org/10.1016/0304-3991(96)00020-4.
